# Editorial: Autoimmunity and the Brain: Paraneoplastic Neurological Injury and Beyond

**DOI:** 10.3389/fneur.2022.900130

**Published:** 2022-05-12

**Authors:** John E. Greenlee, Noel G. Carlson, Justin R. Abbatemarco, Ida Herdlevær, Stacey L. Clardy, Christian A. Vedeler

**Affiliations:** ^1^Neurology Service, George E. Wahlen Veterans Affairs Health Care System, Salt Lake City, UT, United States; ^2^Department of Neurology, University of Utah, Salt Lake City, UT, United States; ^3^George E. Wahlen Veterans Affairs Health Care System, GRECC, Salt Lake City, UT, United States; ^4^Department of Neurobiology, University of Utah, Salt Lake City, UT, United States; ^5^Mellen Center for Multiple Sclerosis Treatment and Research, Cleveland Clinic Foundation, Neurological Institute, Cleveland, OH, United States; ^6^Department of Neurology, Neuro-SysMed, Haukeland University Hospital, Bergen, Norway; ^7^Department of Clinical Medicine, University of Bergen, Bergen, Norway

**Keywords:** autoimmune neurology, autoimmune encephalitis, paraneoplastic neurological syndromes, tissue culture, animal models, immune checkpoint inhibitors, treatment

Autoimmune encephalitides—including the paraneoplastic disorders associated with malignancy —have come to be recognized as major and surprisingly common causes of potentially treatable neurological disease. The disorders, first thought to be rare autoimmune complications of cancer, are now recognized to occur in individuals with and without cancer and to involve antibodies directed against antigens found either at the neuronal cell surface or present in the neuronal cytoplasm or nucleus. Today, we know that conditions associated with antibodies to cell surface antigens may be paraneoplastic or non-paraneoplastic, are usually associated with non-lethal neuronal dysfunction, and are frequently treatable. In contrast, conditions associated with antibodies to intracellular neuronal proteins are usually found in patients with underlying systemic cancer, are characterized by neuronal death, and tend to respond poorly to treatment. At present, over 50 separate antineuronal antibodies have been associated with these disorders, with additional potentially clinically relevant autoantibodies being described every year.

This Research Topic in Frontiers in Neurology addresses several aspects of autoimmune and paraneoplastic encephalitides as understood at this point in time. These include a review of pathogenesis (Greenlee et al.), discussion by Fredrich et al. and Ruiz-Garcia et al. of antibody testing in the diagnosis of these conditions in clinical practice; an intriguing case report by Bartley et al. regarding the use of phage display library in identifying anti-Yo antibody not detectable by immunofluorescence; characterization of antibody-mediated neurological syndromes by Garza and Piquet, Totland et al., and Liu et al.; articles concerning the use of biomarkers in studying these conditions as well as neuromyelitis spectrum disorders by Li Q. et al., Kammeyer et al., Mizenko et al., Chen et al., and Li Y. et al.; and a review by Zoccarato et al. of the important but less-studied area of paraneoplastic neuropathies. Each of these articles makes a valuable contribution the field.

Despite the rapid growth of knowledge in this field, a number of important questions remain to be answered. The first of these, given the steadily expanding number of identified antineuronal autoantibodies, is the urgent need for timely, effective identification of antibodies which at present may require use of multiple testing panels or evaluations. There is also the question of detecting antibodies not yet available in commercial tests or detecting multiple antibodies present in an individual patient. One possible approach, to facilitate early initiation of treatment, could be a uniform system in which laboratories initially reported the presence of antineuronal antibodies, followed by subsequent identification of the antineuronal antibody itself. In this area, the role of the more advanced techniques such as those using phage display analysis remains unstudied; and the report by Bartley et al., which describes phage display detection of anti-Yo antibodies in a case of myelopathy where immunoreactivity to Purkinje cells could not be detected, raises important questions about the use of this technique in diagnosis.

The second of these areas involves pathogenesis, made particularly relevant by the occurrence of autoimmune encephalitides in individuals treated with immune checkpoint inhibitors (Greenlee et al.). Although elegant work by Chefdeville et al. and Small et al. has analyzed expression of NMDAR and CDR2/CDR2L antigens in tumors of patients with anti-NMDAR and anti-Yo antibody responses (1, 2), the sequence of events which leads from tumor expression of antigen to central nervous system involvement has not been delineated. Thus, it is not known why these tumor antigens can provoke a massive immune response at a time when the neoplasm itself is so small as to be undetectable by PET imaging; nor do we actually know the sequence of events which leads to entry of this immune response into the central nervous system and attack on individual neurons. The role of antibodies to neuronal surface proteins such as anti-NMDAR, anti-AMPAR or antibodies to LGI1 and CASPR2 in disease pathogenesis has been extensively studied [Greenlee et al.; (3–5)], and the findings obtained in these studies can almost certainly be extended to other similar autoantibodies. However, the mechanisms of injury identified in studies with these antibodies may or may not be applicable to other antibody-associated syndromes such as the multifocal lesions which occur in GABA_A_ encephalitis or the central and peripheral manifestations associated with antibodies to CRMP5 [Totland et al.; (6)]. Similarly, although Linnoila et al. have developed an animal model to study the NMDAR encephalitis that can follow herpes simplex virus infection (7), it is unclear why some infections, such as herpes simplex virus encephalitis, generate an antineuronal antibody (anti-NMDAR) response, whereas this is not seen in many other infectious and non-infectious processes causing neuronal death.

In contrast to our knowledge concerning antibodies to neuronal surface membrane antigens, the role of antibodies to intracellular neuronal antigens—such as anti-Yo or anti-Hu—remains controversial. Although both anti-Yo and anti-Hu antibodies from affected patients have been shown to be taken up by neurons *in vitro* and to produce neuronal death (8, 9), antibody-mediated neuronal injury has not been proven in an animal model. It should be noted, however, that the great majority of attempts to develop an animal model have studied anti-Yo antibodies, and that virtually all of these experiments have immunized animals with proteins or DNA encoding the Yo antigen, CDR2 (Greenlee et al.). This is important because recent work strongly suggests that the pathogenic antigen involved in anti-Yo antibody response is not CDR2, as has long been assumed, but, rather, the related protein, CDR2L (10); and attempts to produce an animal model using CDR2L have not been reported. Similar concerns exist concerning the role of T lymphocytes in pathogenesis. Although brains of affected patients with anti-Yo or anti-Hu antibodies often contain infiltrates of cytotoxic T lymphocytes, neuronal injury by sensitized T lymphocytes duplicating paraneoplastic disease has also never been demonstrated *in vitro* or in an animal model (Greenlee et al.). An unanswered—and significant—question is how T lymphocytes might target neurons, given that normal adult neurons do not express the MHC class I molecules needed to allow recognition by cytotoxic T cells (11). Neuronal upregulation of MHC class I expression has been described in other clinical and experimental settings (12, 13), but this has not been analyzed in human paraneoplastic neurological disease or in neurons exposed to anti-Yo or anti-Hu antibodies *in vitro* or *in vivo*. A major roadblock to our understanding of disease pathogenesis thus remains the lack of animal models which parallel the natural course of human paraneoplastic and other autoimmune encephalitides seen in humans.

An additional area where our knowledge is inadequate has to do with the pathogenesis of the various categories of peripheral nerve injury associated with cancer. Subacute sensory neuronopathy was the prototypical paraneoplastic disorder affecting the peripheral nervous system (e.g., anti-Hu and anti-CRMP5). The spectrum of immune mediated neuropathies has greatly expanded over the past decade and now includes neuronal surface antibodies such as CASPR2 (14). Some antibody-associated disorder have both central and peripheral symptoms such as anti-KLHL11 and PCA2 antibodies, further expanding the phenotype of peripheral nervous system disorders (Zoccarato et al.). Additionally, neuropathies associated with antibody to myelin-associated glycoprotein and their role in neuropathies associated with plasma cell dyscrasias need to be further investigated [Zoccarato et al.; (15, 16)].

The final—and clinically most important—area has to do with treatment of affected patients. Although several authors such as Graus et al. and Abboud et al. have published excellent clinical guidelines for provisionally diagnosing these disorders and initiating immunomodulatory therapies (16, 17), treatment of these disorders remain empiric, without controlled trials to guide the use of modalities including corticosteroids, immunoglobulin G (IgG), plasma exchange, or agents such as rituximab. In this regard, the first international, multi-institutional, double-blind NIH NeuroNext trial (NN111, ExTINGUISH Trial, ClinicalTrials.gov: NCT04372615) involving the CD19-specific monoclonal, inebilizumab, in NMDAR encephalitis represents an exciting and important step forward. Badly needed—for patients with antibodies to neuronal surface antigens as well as patients with antibodies to intracellular neuronal antigens—are actual studies, using standardized protocols involving the agents currently in use, such as corticosteroids, IVIG, PLEX, or rituximab singly or in combination. Such studies would be difficult to fund but could conceivably be carried out over time on a less formal multi-institutional basis and, like use of pre-clinical animal models, could provide invaluable information regarding treatment. Future studies also need to explore and validate more robust clinical outcomes measures beyond the modified Rankin scale, which is heavily weighted toward motor deficits and does not encompass cognitive impairment and psychiatric/behavioral sequelae seen in frequently seen in patients with autoimmune encephalitis. These future scoring systems could even help identify patients who may benefit from more aggressive/longer duration immunotherapy or more meaningful outcomes such as resumption of gainful employment or schooling.

The decade ahead promises to be fascinating in terms of advancement of knowledge and development of new diagnostic and therapeutic approaches for this important group of disorders.

## Author Contributions

JG, NC, SC, and CV conceived and wrote the initial draft of this manuscript. JA and IH contributed to the final revision as submitted. All authors contributed to the article and approved the submitted version.

## Funding

This work was supported by a Merit Review Award from the United States Department of Veterans Affairs (JG) awards from the Western Institute for Biomedical Research (JG and SC) and by grants from Helse Vest (CV).

## Conflict of Interest

The authors declare that the research was conducted in the absence of any commercial or financial relationships that could be construed as a potential conflict of interest.

## Publisher's Note

All claims expressed in this article are solely those of the authors and do not necessarily represent those of their affiliated organizations, or those of the publisher, the editors and the reviewers. Any product that may be evaluated in this article, or claim that may be made by its manufacturer, is not guaranteed or endorsed by the publisher.
